# Symptom burden correlates to impairment of diffusion capacity and exercise intolerance in long COVID patients

**DOI:** 10.1038/s41598-022-12839-5

**Published:** 2022-05-25

**Authors:** Johannes Kersten, Alexander Wolf, Luis Hoyo, Elina Hüll, Marijana Tadic, Stefanie Andreß, Sascha d’Almeida, Dominik Scharnbeck, Eva Roder, Petra Beschoner, Wolfgang Rottbauer, Dominik Buckert

**Affiliations:** 1 Department for Internal Medicine II, University Hospital Ulm, University of Ulm, Albert-Einstein-Allee 23, 89081 Ulm, Germany; 2 Department of Psychosomatic Medicine and Psychotherapy, University of Ulm, Ulm, Germany

**Keywords:** Cardiology, Diseases, Health care, Signs and symptoms

## Abstract

After acute infection with the SARS-CoV-2 virus, a considerable number of patients remains symptomatic with pathological changes in various organ systems. This study aimed to relate the physical and mental burden of symptoms of long COVID patients to the findings of a somatic evaluation. In patients with persistent long COVID symptoms three months after acute infection we assessed physical and mental health status using the SF-36 questionnaire. The cohort was dichotomised by the results (upper two quartiles vs. lower to quartiles) and compared with regard to transthoracic echocardiography, body plethysmography (including diffusion capacity), capillary blood gas analysis and 6-min walk test (6-MWT). From February 22 to September 13, 2021, 463 patients were prospectively examined, of which 367 completed the SF-36 questionnaire. A positive correlation between initial disease severity (need for hospitalization, intensive care medicine) and resulting symptom burden at follow-up could be demonstrated. Patients with impaired subjective physical and mental status were significantly more likely to be women. There was a significant correlation between symptom severity and reduced exercise tolerance in the 6-MWT (495.6 ± 83.7 m vs 549.7 ± 71.6 m, p < 0.001) and diffusion capacity for carbon monoxide (85.6 ± 14.3% of target vs 94.5 ± 14.4, p < 0.001). In long COVID patients, initial disease severity is correlated with symptom burden after at least 3 months of follow-up. Highly symptomatic long COVID patients show impaired diffusion capacity and 6-MWT despite average or mildly affected mechanical lung parameters. It must be further differentiated whether this corresponds to a transient functional impairment or whether it is a matter of defined organ damage.

## Introduction

Even after 3 months of a healed COVID-19 illness, many patients still experience persistent symptoms with a heterogeneous pattern consisting mainly of respiratory and neuropsychological complaints. Still, otolaryngological, endocrinological and dermatological symptoms are frequently reported^1–3^. The most common symptoms are dyspnoea and fatigue (“head fog”). These are particularly impairing for patients in everyday life. Given the ongoing pandemic, structured studies of this patient collective are essential.

Potential mechanisms leading to long COVID are the persistence of viruses or virus components, autoimmunological processes, metabolic and endocrinological dysregulations, psychosocial factors, microvascular and mitochondrial dysfunction. Long-lasting sequalae have already been described after severe illnesses^4,5^ or after infections with some distinct pathogens^6,7^. Different organic changes in the context of long COVID are described with proposed diagnostic workups from different societies^8–10^. The real impact of the sometimes only minor organic changes on the ethiological assignment of the complaints is often limited.

Given the large number of patients recovered from COVID-19, long COVID is a problem that cannot yet be fully grasped. Therefore, performing a senseful risk stratification is essential to identify patients at risk of permanent health impairment and address it diagnostically and therapeutically. A direct association between diagnostics and the variously expressed subjective complaints seems possible, to a limited extent, in daily practice. Therefore, this study aimed to correlate the physical and mental symptom burden of long COVID patients with their actual findings from the somatic evaluation.

## Methods

Consecutive patients who presented to the specialized long COVID unit of our university tertiary care centre were included. Patients presented on their own initiative or as a referral from their general practitioner. All patients had a SARS-CoV-2 infection detected by polymerase chain reaction at least three months before their visit. All patients were included independently of their initial disease severity (ranging from asymptomatic to hospitalised courses). As recently published, diagnostic workups followed a strict examination algorithm^11^. In brief, all patients underwent transthoracic echocardiography, body plethysmography, capillary blood gas analysis (BGA) and a 6-min walk test (6-MWT). This is intended to narrow down or exclude common causes of cardiopulmonary symptoms using widely available basic diagnostic tools. In the case of pathological changes, further diagnostics are indicated, while relevant pathologies can be ruled out in the case of inconspicuous results. This approach, focusing on basic diagnostics, is intended to address the high number of affected individuals and the often expensive and more complex additional diagnostics such as cardiopulmonary exercise testing and cardiopulmonary magnetic resonance imaging.

Also, a survey was carried out using the standardised SF-36 questionnaire to obtain a somatic assessment of the patients (version 1.0)^12^. This questionnaire consists of eight dimensions intended to depict the physical and mental well-being of subjects: general health, physical functioning, role limitations owing to physical health, role limitations owing to emotional problems, energy/fatigue, emotional well-being, social functioning and pain. The dimensions were summarised in the superordinate variables physical component summary (PCS) and mental component summary (MCS)^13^. The arithmetic mean from the individual physical measuring scales of the SF-36 questionnaire was calculated (general health, physical functioning, role limitations owing to physical health and pain) to determine PCS as a measurement of subjective physical well-being. MCS as a tool for evaluating subjective mental health was calculated analogously as the mean of the psychological parameters (role limitations owing to emotional problems, energy/fatigue, emotional well-being and social functioning).

The study was approved by the ethics committee of the University of Ulm (approval number 406/20) and conducted in accordance with the principles of the Declaration of Helsinki. Patients or the public were not involved in the design, or conduct, or reporting, or dissemination plans of our research.

### Statistics

For descriptive analysis, continuous variables were expressed as means ± standard deviations, and categorical values were expressed as numbers and percentages. The study group was dichotomised by the median. Thereby, a group with better PCS (above the median) was compared with patients below the median and with MCS accordingly. The Student’s t-test was used to compare means in continuous variables. Comparisons of categorical variables were performed using the chi-square test. An ANOVA analysis was performed with a post-hoc test (Bonferroni) as appropriate to compare different courses of initial COVID-19. To compare the somatic examination findings grouped according to physical or mental self-perception, a supplementary grouped evaluation according to initial disease severity was performed as an unpaired t-test using bootstrapping (number of samples 1000). A two-tailed p-value of < 0.05 was considered statistically significant for every test except in the case of multiple testing in the post-hoc tests, where an alpha level of < 0.01 was used. Analyses were performed using IBM SPSS Statistics 26 (IBM, Armonk, NY, USA).

### Ethics approval

The study was approved by the ethics committee of the University of Ulm (approval No. 406/20).

### Consent to participate

Every participant gave written informed consent.

### Consent for publication

All authors have actively participated in this work, reviewed the final draft, and consented to its publication. This manuscript has not been published in part or in its entirety and is not under consideration for publication in any other journal. No portion of the text has been copied verbatim from any sources, and all information provided is accompanied by appropriate references. We are aware that it is the authors’ responsibility to obtain permission for any figures or tables reproduced from previous publications prior to the final acceptance of the manuscript and to fully cover any costs involved. The authors have no conflicts of interest to declare.

## Results

**Figure 1:**
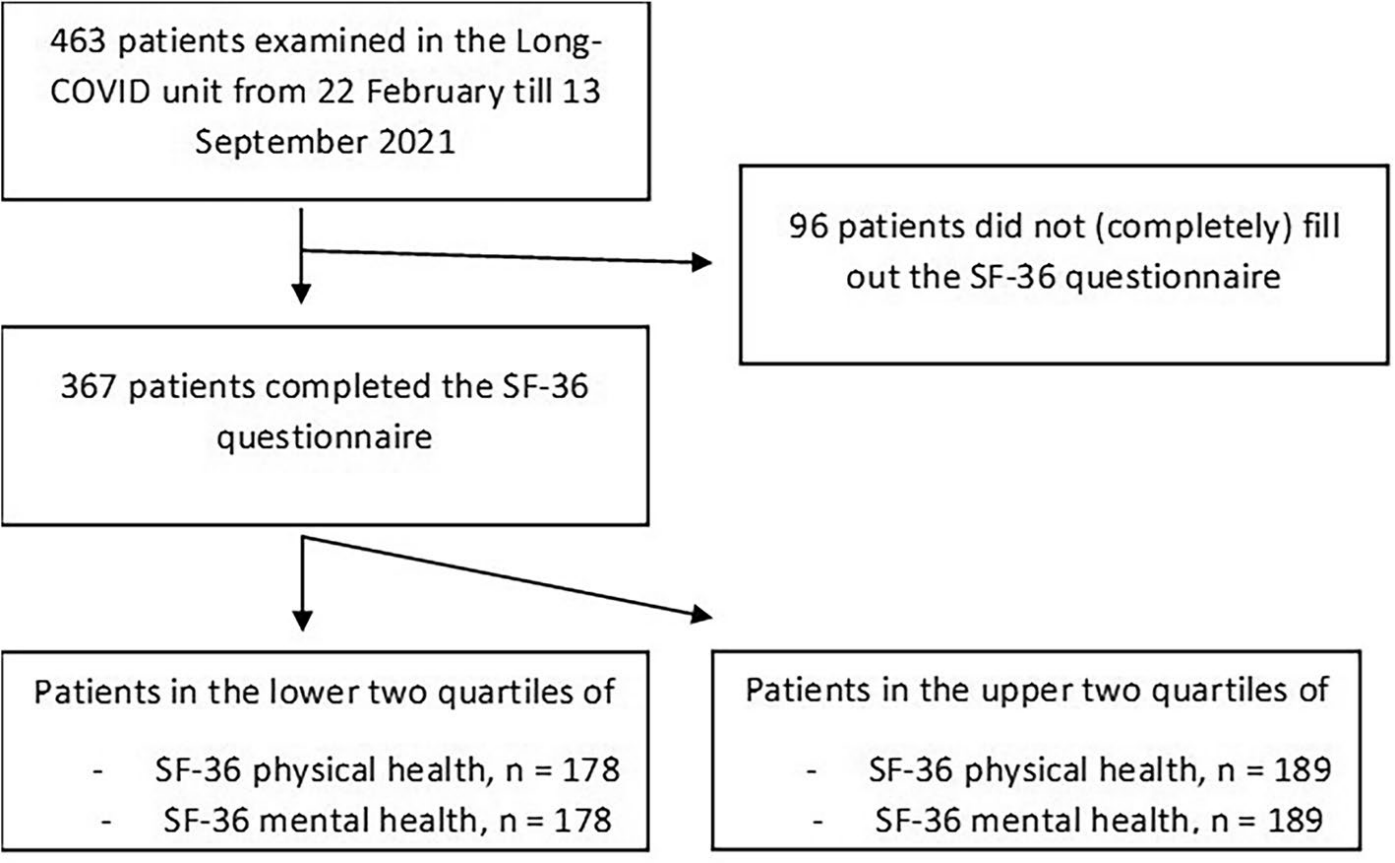
Patient enrolment. Shown is the full study population and the patients who had to be excluded due to missing SF-36 questionnaire. The resulting collective was dichotomized by the median of the physical component summary (median 70.6) and the mental component summary (median 69.1), respectively.

From February 22 to September 13, 2021, 463 patients were prospectively examined. Three hundred sixty-seven had filled out the questionnaire in full and were available for further analysis (see Fig. 1). The cohort was 47.3 ± 14.8 years old, and 57.5% were women. The most common symptoms at the time of presentation in our long COVID unit were fatigue (51.1%) and dyspnoea (42.5%). The remaining baseline characteristics can be seen in Table 1.

**Table 1:** Patient characteristics (n = 367).

Characteristics	Value
Age (years)	47.3 ± 14.8
Women, n (%)	211 (57.5)
Body mass index (kg/m^2^)	25.8 ± 4.8
**Patient history**	
Cardiac diseases, n (%)	21 (5.7)
Pulmonary diseases, n (%)	44 (12.0) [asthma bronchial, 33 (8.2)]
Malignant diseases, n (%)	10 (2.7)
**Cardiovascular risk profile**	
Arterial hypertension, n (%)	76 (20.7)
Diabetes mellitus type I, n (%)	3 (0.8)
Diabetes mellitus type II, n (%)	13 (3.5)
Dyslipidaemia, n (%)	192 (52.3)
Current/past smoking, n (%)	68 (18.6)
**Long COVID symptoms**	
Thoracic pain/pressure, n (%)	75 (20.5)
Dyspnoea, n (%)	156 (42.5)
Anosmia/ageusia, n (%)	37 (10.1)
Headaches, n (%)	32 (8.7)
Sleep disorders, n (%)	33 (9.1)
Exhaustion/fatigue, n (%)	187 (51.1)
Memory and concentration disorders, n (%)	90 (24.5)

Continuous variables are expressed as means ± standard deviations. Categorical variables are expressed as numbers (percentages).

The values for the eight SF-36 questionnaire categories are shown in Table 2. MCS was slightly but significantly lower than the PCS (64.2 ± 22.4 vs. 67.1 ± 22.6, p < 0.001). The initial illness severity from COVID-19 was associated with the intensity of long COVID symptoms. Each dimension of SF-36 and MCS and PCS were significantly different between hospitalised, symptomatic and asymptomatic/oligosymptomatic courses of the initial COVID-19 disease except for “pain” (Table 2) which was different only in trend. Differences between hospitalised and initially asymptomatic/oligosymptomatic patients remained statistically significant in a post-hoc Bonferroni test. Despite COVID-19 severity, corticosteroid medication was associated with symptom severity in PCS but not in MCS. Patients with corticosteroid intake during COVID-19 had worse PCS than patients without it (8.5% vs 2.6%, p = 0.014). In addition to symptom burden, several examination findings were associated with initial symptom severity. In particular, worse values for total lung capacity, forced vital capacity (FVC), and DLCO were found in patients with initially more severe COVID-19 disease, as can be seen in Table 2.

**Table 2:** Results of SF-36 evaluation and results of transthoracic echocardiography, 6-min walk test, body plethysmography and capillary blood gas test in dependency of initial disease severity.

SF-36 category	Hospitalised COVID-19 (n = 25)	Symptomatic COVID-19 (n = 295)	Asymptomatic/oligosymptomatic COVID-19 (n = 47)	p-value
**Physical component summary**	**53.4 ± 23.7**	**66.5 ± 22.2**	**77.7 ± 19.9**	**< 0.001**
General health	49.2 ± 19.6	58.9 ± 18.6	66.1 ± 19.8	**0.001**
Physical functioning	61.6 ± 24.7	76.6 ± 21.6	86.8 ± 17.8	**< 0.001**
Role limitations owing to physical health	38.0 ± 44.6	57.1 ± 39.9	77.7 ± 24.3	**< 0.001**
Pain	64.6 ± 25.5	73.3 ± 27.1	80.3 ± 22.5	0.052
**Mental component summary**	**53.7 ± 24.3**	**63.5 ± 22.2**	**74.2 ± 19.1**	**< 0.001**
Role limitations owing to emotional problems	45.3 ± 44.0	68.5 ± 39.4	83.0 ± 30.2	**0.001**
Energy/fatigue	41.8 ± 21.9	45.2 ± 20.4	55.2 ± 21.9	**0.005**
Emotional well-being	63.8 ± 17.3	67.4 ± 18.4	76.5 ± 15.6	**0.003**
Social functioning	64.0 ± 29.2	72.8 ± 27.3	82.2 ± 24.7	**0.018**
**Transthoracic echocardiography**				
Left ventricular ejection fraction (%)	58.1 ± 8.2	60.0 ± 6.7	61.3 ± 8.4	0.381
Left ventricular global longitudinal strain (%)	− 17.1 ± 3.5	− 18.5 ± 2.6	− 17.8 ± 2.7	0.196
**6-min walk test**				
Distance (m)	497.3 ± 90.8	523.8 ± 81.1	536.0 ± 84.0	0.176
Borg dyspnoea scale (at the end)	3.9 ± 2.2	2.9 ± 2.0	1.8 ± 1.9	**< 0.001**
Borg exertion scale (at the end)	2.9 ± 2.7	2.4 ± 2.1	1.8 ± 1.7	0.079
**Body plethysmography**				
Total lung capacity, % of target	96.8 ± 16.3	104.1 ± 14.0	106.6 ± 12.4	**0.020**
Residual volume, % of target	104.2 ± 27.4	116.3 ± 23.9	118.3 ± 27.0	0.053
Forced vital capacity, % of target	90.7 ± 15.1	93.2 ± 12.1	97.8 ± 12.0	**0.030**
FEV1, % of target	94.4 ± 16.6	95.4 ± 13.6	100.0 ± 13.4	0.092
Tiffeneau-Index, % of target	104.0 ± 9.5	102.3 ± 7.4	101.8 ± 7.0	0.477
Diffusion capacity for CO, % of target	79.6 ± 19.3	90.3 ± 14.2	95.2 ± 14.3	**< 0.001**
**Capillary blood gas test**				
pO_2_ (mmHg)	77.8 ± 9.3	78.0 ± 9.2	78.9 ± 8.6	0.834
pCO_2_ (mmHg)	35.8 ± 3.6	36.8 ± 3.8	37.9 ± 7.2	0.124

Variables are expressed as means ± standard deviations.

Significant values are in [bold].

Patients in the two lower quartiles in PCS tended to be older, female, with a higher body mass index and have hypertension (Table 3). There was an impairment of functional capacity in the meaning of a reduced walking distance in the 6-MWT (495.6 ± 83.7 m vs 549.7 ± 71.6 m, p < 0.001) and higher values on the Borg scale for dyspnoea (3.8 ± 2.0 vs 2.0 ± 1.7, p < 0.001) and exertion (3.5 ± 2.2 vs 1.4 ± 1.3, p < 0.001). In body plethysmography, the forced vital capacity and diffusion capacity for carbon monoxide (DLCO) were significantly worse in the group with impaired physical health in the self-estimation by the SF-36 questionnaire (FVC: 92.1 ± 12.9% of target vs 94.9 ± 11.7% of target, p = 0.030; DLCO: 85.6 ± 14.3% of target vs 94.5 ± 14.4, p < 0.001). There were no differences in the other ventilatory volumes. A significant but in absolute terms only slight difference was also seen in blood gases with lower values for oxygen and carbon dioxide in the group with lower PCS.

**Table 3:** Cardiac and pulmonary function depending on the assessment of physical health by the SF-36 questionnaire.

Characteristic	Impaired physical health (n = 178)	Less impaired physical health (n = 189)	p-value
Age (years)	49.5 ± 14.1	45.1 ± 15.1	**0.004**
Women, n (%)	122 (68.5)	89 (47.1)	**< 0.001**
Body mass index (kg/m^2^)	26.6 ± 5.2	25.0 ± 4.3	**0.001**
Hypertension, n (%)	45 (25.3)	31 (16.4)	**0.036**
Type 2 diabetes, n (%)	7 (3.9)	6 (3.2)	0.695
Smoking, n (%)	37 (20.8)	31 (16.4)	0.291
**Patient history**			
Cardiac diseases, n (%)	11 (6.2)	21 (11.1)	0.684
Pulmonary diseases, n (%) [asthma bronchial, n (%)]	25 (14.0) [21 (11.8)]	19 (10.1) [12 (6.3)]	0.224
Malignant diseases, n (%)	7 (3.9)	3 (1.6)	0.159
Time since end of quarantine (days)	168.6 ± 99.3	190.5 ± 109.1	**0.045**
**COVID-19 history**			
Oligosymptomatic/asymptomatic course, n (%)	12 (6.7)	35 (18.5)	**< 0.001**
Hospitalisation, n (%)	17 (9.6)	8 (4.2)	**0.043**
Invasive ventilation, n (%)	6 (3.4)	2 (1.1)	0.129
Therapy with corticosteroids, n (%)	15 (8.5)	5 (2.6)	**0.014**
Therapy with antibiotics, n (%)	13 (7.3)	9 (4.8)	0.305
**Transthoracic echocardiography**			
Left ventricular ejection fraction (%)	59.5 ± 6.7	60.6 ± 7.2	0.248
Left ventricular global longitudinal strain (%)	− 18.2 ± 2.7	− 18.4 ± 2.7	0.584
**6-min walk test**			
Distance (m)	495.6 ± 83.7	549.7 ± 71.6	**< 0.001**
Borg dyspnoea scale (at the end)	3.8 ± 2.0	2.0 ± 1.7	**< 0.001**
Borg exertion scale (at the end)	3.5 ± 2.2	1.4 ± 1.3	**< 0.001**
**Body plethysmography**			
Total lung capacity, % of target	102.8 ± 15.3	105.0 ± 12.8	0.138
Residual volume, % of target	114.9 ± 23.8	116.5 ± 25.4	0.526
Forced vital capacity, % of target	92.1 ± 12.9	94.9 ± 11.7	**0.030**
FEV1, % of target	94.9 ± 13.4	96.9 ± 14.2	0.155
Tiffeneau-Index, % of target	82.1 ± 6.3	82.4 ± 6.7	0.659
Diffusion capacity for CO, % of target	85.6 ± 14.3	94.5 ± 14.4	**< 0.001**
**Capillary blood gas test**			
pO_2_ (mmHg)	76.9 ± 10.2	79.2 ± 7.8	**0.016**
pCO_2_ (mmHg)	36.2 ± 4.4	37.5 ± 4.3	**0.007**
**Blood test**			
Haemoglobin, mean (SD), g/dL [normal, 12.3–15.3]	14.1 ± 1.1	14.1 ± 1.1	0.052
Glomerular filtration rate, mean (SD), mL/min	91.1 ± 16.8	94.1 ± 16.1	0.091
C-reactive protein, mean (SD), mg/L [normal, < 5.0]	2.7 ± 6.9	2.0 ± 5.9	0.284
Thyroid-stimulating hormone, mean (SD), mU/L [normal, 0.400–3.770]	1.7 ± 0.9	1.9 ± 1.6	0.173
D-dimers, mean (SD), mg/L FEU [normal, < 0.50]	0.29 ± 0.22	0.24 ± 0.09	**0.005**
Troponin T, mean (SD), ng/L [normal, < 15.0]	5.0 ± 3.9	5.0 ± 2.4	0.930
NT-proBNP, mean (SD), pg/mL [normal, < 130.0]	78.2 ± 57.5	71.7 ± 92.9	0.429

The lower two quartiles (< 70.6 points) were described as impaired, and the upper two quartiles (> 70.6 points) as less impaired physical health.

Continuous variables are expressed as means ± standard deviations. Categorical variables are expressed as numbers (percentages).

*FEV1* forced expiratory volume in one second, *CO* carbon monoxide, *FEU* fibrinogen-equivalent units, *NT-proBNP* N-terminal pro b-type natriuretic peptide.

Significant values are in [bold].

The differences described were also found grouped by initial disease course, although some results were not statistically significant most likely because of the small subgroups and a high scattering rate. For example, reduced walking distance in the 6-MWT was seen in initially hospitalized patients (485.7 ± 100.1 m vs 525.0 ± 74.4 m, p = 0.342) and outpatients (495.9 ± 79.3 m vs 552.6 ± 72.1 m, p = 0.001) or in asymptomatic/oligosymptomatic patients (514.7 ± 120.2 m vs 542.9 ± 69.6 m, p = 0.453). This was similar in DLCO, where initially hospitalized and asymptomatic/oligosymptomatic patients had numerically worse outcomes in the lower two quartiles of PCS (74.7 ± 17.1% of target vs 88.8 ± 74.7% of target, p = 0.118; and 92.7 ± 19.6% of target vs 96.0 ± 12.0% of target, p = 0.574). Symptomatic outpatients had significantly lower DLCO scores in the two lower quartiles of PCS (86.3 ± 13.2% of target vs 94.5 ± 14.5% of target, p = 0.001).

According to the MCS, similar results were found in the classification (Table 4). There were predominantly women in the more restricted group (69.1% vs 46.6%, p < 0.001). Again, walking distance (502.4 ± 73.3 m vs 543.3 ± 85.2, p < 0.001) and values on the Borg scale were significantly lower in the group with lower MCS. There was a significant difference in DLCO (87.1 ± 16.4% of target vs 93.1 ± 12.9% of target, p < 0.001) and, to a lesser extent, FVC and FEV1. Grouped by initial disease severity, the more restricted group again had worse results. This is reflected in the walking distance in the 6-MWT (hospitalized: 466.7 ± 64.4 m vs 560.6 ± 108.9 m; p = 0.050; symptomatic non-hospitalized: 506.8 ± 70.9 m vs 541.3 ± 86.2 m, p = 0.004; asymptomatic/oligosymptomatic: 505.8 ± 98.5 m vs 548.3 ± 75.7 m, p = 0.125).

**Table 4:** Cardiac and pulmonary function depending on the assessment of mental health by SF-36. The lower two quartiles (< 69.1 points) were described as impaired, and the upper two quartiles (> 69.1 points) as less impaired physical health.

Characteristics	Impaired mental health (n = 178)	Less impaired mental health (n = 189)	p-value
Age (years)	47.2 ± 13.9	47.3 ± 15.6	0.963
Women, n (%)	123 (69.1)	88 (46.6)	**< 0.001**
Body mass index (kg/m^2^)	26.2 ± 5.2	25.4 ± 4.4	0.116
Hypertension, n (%)	40 (22.5)	36 (19.0)	0.418
Type 2 diabetes, n (%)	8 (4.5)	5 (2.6)	0.338
Smoking, n (%)	39 (21.9)	29 (15.3)	0.111
**Patient history**			
Cardiac diseases, n (%)	10 (5.6)	11 (5.8)	0.965
Pulmonary diseases, n (%) [asthma bronchial, n (%)]	23 (12.9) [18 (10.1)]	21 (11.1) [15 (7.9)]	0.566
Malignant diseases, n (%)	6 (3.4)	4 (2.1)	0.444
Time since end of quarantine, d	175.0 ± 100.0	184.4 ± 109.4	0.395
**COVID-19 history**			
Oligosymptomatic/asymptomatic course, n (%)	14 (7.9)	33 (17.5)	**0.006**
Hospitalisation, n (%)	17 (9.6)	8 (4.2)	**0.043**
Invasive ventilation, n (%)	7 (3.9)	1 (0.5)	**0.026**
Therapy with corticosteroids, n (%)	11 (6.2)	9 (4.8)	0.549
Therapy with antibiotics, n (%)	11 (6.2)	11 (5.8)	0.885
**Transthoracic echocardiography**			
Left ventricular ejection fraction (%)	60.2 ± 6.3	59.9 ± 7.6	0.784
Left ventricular global longitudinal strain (%)	− 18.5 ± 2.3	− 18.2 ± 3.0	0.446
**6-min walk test**			
Distance, m	502.4 ± 73.3	543.3 ± 85.2	**< 0.001**
Borg dyspnoea scale (at the end)	3.6 ± 2.1	2.2 ± 1.7	**< 0.001**
Borg exertion scale (at the end)	3.3 ± 2.2	1.6 ± 1.5	**< 0.001**
**Body plethysmography**			
Total lung capacity, % of target	103.1 ± 15.6	104.8 ± 12.4	0.254
Residual volume, % of target	115.3 ± 25.8	116.1 ± 23.5	0.734
Forced vital capacity, % of target	92.1 ± 12.9	94.9 ± 11.7	**0.027**
FEV1, % of target	92.1 ± 12.7	95.0 ± 11.9	**0.021**
Tiffeneau-Index, % of target	82.0 ± 6.8	82.5 ± 6.2	0.317
Diffusion capacity for CO, % of target	87.1 ± 16.4	93.1 ± 12.9	**< 0.001**
**Capillary blood gas test**			
pO_2_, mmHg	77.4 ± 10.0	78.8 ± 8.1	0.137
pCO_2_, mmHg	36.2 ± 4.3	37.5 ± 4.3	**0.005**
**Blood test**			
Haemoglobin, mean (SD), g/dL [normal, 12.3–15.3]	14.2 ± 1.0	14.4 ± 1.1	0.086
Glomerular filtration rate, mean (SD) (mL/min)	92.1 ± 16.6	93.1 ± 16.3	0.578
C-reactive protein, mean (SD) (mg/L) [normal, < 5.0]	2.6 ± 6.8	2.1 ± 6.0	0.410
Thyroid-stimulating hormone, mean (SD) (mU/L) [normal, 0.400–3.770]	1.7 ± 0.9	1.8 ± 1.6	0.472
D-dimers, mean (SD), mg/L FEU [normal, < 0.50]	0.28 ± 0.21	0.25 ± 0.11	0.075
Troponin T, mean (SD), ng/L [normal, < 15.0]	4.7 ± 3.7	5.3 ± 3.7	0.116
NT-proBNP, mean (SD), pg/mL [normal, < 130.0]	75.9 ± 56.7	73.8 ± 93.4	0.796

Continuous variables are expressed as means ± standard deviations. Categorical variables are expressed as numbers (percentages).

*FEV1* forced expiratory volume in one second, *CO* carbon monoxide, *FEU* fibrinogen-equivalent units, *NT-proBNP* N-terminal pro b-type natriuretic peptide.

Significant values are in [bold].

The period since COVID-19 was inversely associated with the severity of symptoms, which was only statistically significant in the PCS classification (p = 0.045). The evaluation of laboratory tests showed significantly higher values for D-dimers in the group with more symptoms of PCS. There was only a trend without significance in the MCS. Values for haemoglobin, glomerular filtration rate, high sensitive troponin t and NT-proBNP showed no relevant differences between groups (Tables 3 and 4).

Evaluation of cardiac function by transthoracic echocardiography revealed no differences between the groups, as defined by the SF-36 assessment. Patients with impaired (physical and mental) health showed the same values for left ventricular ejection fraction (LVEF) and left ventricular global longitudinal strain (LV GLS) as patients with good health in the self-assessment.

## Discussion

The main findings of this study are as follows: (1) the severity of the initial COVID-19 disease and female sex are associated with higher symptom burden in the context of long COVID; (2) the extent of physical and mental impairment in the self-assessment using SF-36 as well as the initial disease severity correlate significantly with DLCO and distance in the 6-MWT and (3) there was no correlation of symptom burden to markers of the left ventricular function in transthoracic echocardiography.

A predominance of the female gender has been described in other long COVID cohorts^1,14^. Immunological and psychological causes were discussed, with a potential overlap. Under the assumption of T-cell abnormalities or autoantibodies leading to long COVID, the reported higher number of T-cells and autoantibodies during and shortly after COVID-19 could be potentially causative for ongoing symptoms^15,16^. On the other hand, women are more prone to depression and anxiety disorders because of their higher oestrogen levels^17–19^. Since the causes of a long COVID syndrome have not yet been fully understood and a multi-causality is likely, the female predominance cannot be adequately explained.

A positive correlation of pulmonary restriction in follow-up with COVID-19 severity was described earlier, especially in hospitalised patients^20,21^. Data on mild or even asymptomatic courses are scarce. In a study carried out in Wuhan by Huang et al., 1733 patients were examined six months after the reduced walking distance in the 6-MWT, and diffusion impairment dependent on the in-hospital course of the initial COVID-19 disease has been shown during hospital discharge^22^. In another trial with hospitalised patients, a DLCO reduction could be associated with female sex and radiological abnormalities in follow-up examinations after 3–12 months^21^. In contrast, our cohort consisted of all clinical courses, including outpatient and asymptomatic, with a mean follow-up time of six months. We could show a correlation of well-being to functional values of diffusion testing and the 6-MWT. While neuropsychological factors can influence symptoms during the 6-MWT and walking distance, the reduction in DLCO suggests an at least transient organic correlate. Whether the numerical differences in DLCO, some of which are small, are actually causal for the patients' dyspnea remains questionable. Patients with a low DLCO can be rehabilitated well and improvements in DLCO and 6-MWT go hand in hand^23^. Therefore, our results are of clinical relevance, as they illustrate that rehabilitative therapy of dyspnea and exercise capacity also improves patients' self-assessed physical and mental health; thus, no separate therapeutic approach is necessary for this.

In the longitudinal trial by Steinbeis et al., an improvement in respiratory function has been shown, including DLCO over time^20^. Considering the possible time course of DLCO and the significantly different observation times of the cohorts after COVID-19 infection in our collective, a possible confounder or coincidence is possible here.

In another trial, a time dependency of symptom burden in the context of long COVID has been shown in 13.3% of participants with symptoms lasting more than 28 days after COVID-19 and only 2.3% for more than 12 weeks^1^. This is in line with the assumption that most cases of long COVID are another form of post-infectious syndromes, which have also shown time dependency^7,24,25^. It currently remains unclear to what extent an improvement in lung function can be achieved and by which measures this process can be positively influenced. In a synopsis of the achievable final state and, if necessary, further imaging examinations, a distinction can ultimately be made between functional impairment (“functional” long COVID) and actual organ damage as an expression of a distinct disease (e.g. myocarditis, pulmonary embolism, pulmonary restriction). In this context, the increased levels of D-dimers in comparison between more symptomatic and less symptomatic patients may be of interest. These findings are potential indications of prothrombotic conditions, possibly associated with an increased incidence of pulmonary embolism. However, this condition is only suggestive and should be investigated in further studies.

Long COVID is affecting patients regardless of the initial severity of the disease^26^. It is pointed to a missing or even inverse relationship between COVID-19 severity and the ongoing symptoms in some studies. No differences in symptom burden and walking distance in the 6-MWT between different clinical courses of COVID-19 have been found in a study by Townsend et al.^27^. There was a relevant selection bias risk with only 153 out of 487 patients (31%) who accepted the offer for the examination in their trial. In return, there is also the possibility of selection bias in our setting of a long COVID unit. There were apparent differences between the self-assessed symptom severity in our cohort of previously hospitalised, outpatient and asymptomatic/oligosymptomatic patients. Initial disease severity was further associated with changes in body plethysmography (specifically total lung capacity, FVC, and DLCO) in our study. From this, it could be inferred that the association of symptom burden and functional parameters would be rather coincidental in nature. Since patients with higher self-assessed symptom severity also had worse outcomes in the subgroups according to initial disease severity, we assume a combined effect. Because the cohorts of initially hospitalized and asymptomatic/oligosymptomatic patients are very small, our results in these groups can only be considered hypothesizing. Cardiac inflammation signs as part of post-COVID-19 sequelae were frequently reported earlier^28–30^. Most of them showed elevated levels of markers for inflammation in non-invasive tissue characterisation using cardiovascular magnetic resonance (CMR) imaging (T1, T2 and ECV). It was unclear whether these data were attributable to long-lasting symptoms after COVID-19. In our cohort, we found no correlation between more severe symptoms and LVEF or LV GLS. In another trial by Joy et al., CMR scans were carried out in seropositive and seronegative healthcare workers, and no differences in markers for inflammation were found. Since there was only one hospitalised patient because of COVID-19 in their cohort, cardiac involvement or impairment is possibly correlated with the severity of the initial COVID-19 course. Elevated markers of myocardial inflammation with normalisation in a follow-up visit have been shown in another trial with 58 hospitalised patients (36.1% admission to intensive care unit)^31^. The researchers also carried out functional lung tests, blood samples and cardiopulmonary exercise tests, finding no correlation to the symptoms of the patients. The difference in our data may be a consequence of COVID-19 severity or the fact that symptoms were assessed categorically rather than gradually in our study. Interestingly, they found a significant difference in right ventricular ejection fraction and volume between baseline and a follow-up visit, with an improvement over time, unfortunately not evaluated in our data. This could be a goal for further investigation as the right ventricular strain is easy to assess by transthoracic echocardiography^32^.

## Conclusion

A correlation of long COVID symptoms with the severity of the course of the initial COVID-19 disease and cardiopulmonary function markers is suggested by our data, particularly DLCO and the 6-MWT. A longer-lasting residual due to a more severe COVID course appears likely. Conversely, cardiopulmonary diagnostics do not appear necessary in low-symptomatic initial COVID-19 courses or subjectively less impaired patients. Whether the worsening of DLCO or walking distance in the 6-MWT is causal or coincidental for the symptoms needs to be investigated in further studies. Myocardial injury resulting in a reduction of left ventricular function is not correlated with symptoms of long COVID.

## Limitations

Selection bias is likely for our investigation because patients were referred to us on their initiative or from a general practitioner. This leads to the assumption that patients with mild and acute symptoms of long COVID were less likely to come to our unit. A study of larger or further prespecified cohorts would provide the opportunity to make more differentiated statements about changes in diagnostics. In particular, the groups with initially hospitalized and asymptomatic/oligosymptomatic patients were too small to provide reliable results in our study.

## Data Availability

The datasets used and/or analyzed during the current study are available from the corresponding author on reasonable request.

## Abbreviations

6-MWT: 6-Minute walk test
CMR: Cardiovascular magnetic resonance
CRP: C-reactive protein
DLCO: Diffusing capacity of the lungs for carbon monoxide
FEU: Fibrinogen**-**equivalent units
FEV1: Forced expiratory volume in the first second
FVC: Forced expiratory capacity
LVEF: Left ventricular ejection fraction
LV GLS: Left ventricular global longitudinal strain
MCS: Mental component summary (of the SF-36)
NT-proBNP: N-terminal pro b-type natriuretic peptide
pCO2: Partial pressure of carbon dioxide
PCS: Physical component summary (of the SF-36)
pO2: Partial pressure of oxygen

## References

[1] Sudre CH, Murray B, Varsavsky T, Graham MS, Penfold RS, Bowyer RC (2021). Attributes and predictors of long COVID. Nat. Med..

[2] Nalbandian A, Sehgal K, Gupta A, Madhavan MV, McGroder C, Stevens JS (2021). Post-acute COVID-19 syndrome. Nat. Med..

[3] Bakılan F, Gökmen İG, Ortanca B, Uçan A, Eker Güvenç Ş, Şahin Mutlu F (2021). Musculoskeletal symptoms and related factors in postacute COVID-19 patients. Int. J. Clin. Pract..

[4] Kress JP, Hall JB (2014). ICU-acquired weakness and recovery from critical illness. N. Engl. J. Med..

[5] Pandharipande PP, Girard TD, Jackson JC, Morandi A, Thompson JL, Pun BT (2013). Long-term cognitive impairment after critical illness. N. Engl. J. Med..

[6] Shikova E, Reshkova V, Kumanova A, Raleva S, Alexandrova D, Capo N (2020). Cytomegalovirus, Epstein-Barr virus, and human herpesvirus-6 infections in patients with myalgic encephalomyelitis/chronic fatigue syndrome. J. Med. Virol..

[7] Hickie I, Davenport T, Wakefield D, Vollmer-Conna U, Cameron B, Vernon SD (2006). Post-infective and chronic fatigue syndromes precipitated by viral and non-viral pathogens: Prospective cohort study. BMJ.

[8] Petersen EL, Goßling A, Adam G, Aepfelbacher M, Behrendt C-A, Cavus E (2022). Multi-organ assessment in mainly non-hospitalized individuals after SARS-CoV-2 infection: The Hamburg City Health Study COVID programme. Eur. Heart J..

[9] Raman B, Bluemke DA, Lüscher TF, Neubauer S (2022). Long COVID: Post-acute sequelae of COVID-19 with a cardiovascular focus. Eur. Heart J..

[10] Gluckman TJ, Bhave NM, Allen LA, Chung EH, Spatz ES, Ammirati E (2022). 2022 ACC Expert Consensus Decision Pathway on Cardiovascular Sequelae of COVID-19 in Adults: Myocarditis and other myocardial involvement, post-acute sequelae of SARS-CoV-2 infection, and return to play: A report of the American College of Cardiology Solution Set Oversight Committee. J. Am. Coll. Cardiol..

[11] Kersten J, Baumhardt M, Hartveg P, Hoyo L, Hüll E, Imhof A (2021). Long COVID: Distinction between organ damage and deconditioning. J. Clin. Med..

[12] Hays RD, Sherbourne CD, Mazel RM (1993). The RAND 36-item health survey 1.0. Health Econ..

[13] Ware JE, Kosinski M, Bayliss MS, McHorney CA, Rogers WH, Raczek A (1995). Comparison of methods for the scoring and statistical analysis of SF-36 health profile and summary measures: Summary of results from the Medical Outcomes Study. Med. Care..

[14] Bai F, Tomasoni D, Falcinella C, Barbanotti D, Castoldi R, Mulè G (2021). Female gender is associated with long COVID syndrome: A prospective cohort study. Clin. Microbiol. Infect..

[15] Takahashi T, Ellingson MK, Wong P, Israelow B, Lucas C, Klein J (2020). Sex differences in immune responses that underlie COVID-19 disease outcomes. Nature.

[16] Sukocheva OA, Maksoud R, Beeraka NM, Madhunapantula SV, Sinelnikov M, Nikolenko VN (2021). Analysis of post COVID-19 condition and its overlap with myalgic encephalomyelitis/chronic fatigue syndrome. J. Adv. Res..

[17] Halbreich U, Kahn LS (2001). Role of estrogen in the aetiology and treatment of mood disorders. CNS Drugs.

[18] Jacobi F, Wittchen H-U, Holting C, Höfler M, Pfister H, Müller N (2004). Prevalence, co-morbidity and correlates of mental disorders in the general population: Results from the German Health Interview and Examination Survey (GHS). Psychol. Med..

[19] Marcus SM, Young EA, Kerber KB, Kornstein S, Farabaugh AH, Mitchell J (2005). Gender differences in depression: Findings from the STAR*D study. J. Affect. Disord..

[20] Steinbeis F, Thibeault C, Doellinger F, Ring RM, Mittermaier M, Ruwwe-Glösenkamp C (2021). Severity of respiratory failure and computed chest tomography in acute COVID-19 correlates with pulmonary function and respiratory symptoms after infection with SARS-CoV-2: An observational longitudinal study over 12 months. Respir. Med..

[21] Wu X, Liu X, Zhou Y, Yu H, Li R, Zhan Q (2021). 3-month, 6-month, 9-month, and 12-month respiratory outcomes in patients following COVID-19-related hospitalisation: A prospective study. Lancet Respir. Med..

[22] Huang C, Huang L, Wang Y, Li X, Ren L, Gu X (2021). 6-month consequences of COVID-19 in patients discharged from hospital: A cohort study. Lancet.

[23] Liu K, Zhang W, Yang Y, Zhang J, Li Y, Chen Y (2020). Respiratory rehabilitation in elderly patients with COVID-19: A randomized controlled study. Complement. Ther. Clin. Pract..

[24] Rebman AW, Aucott JN (2020). Post-treatment lyme disease as a model for persistent symptoms in lyme disease. Front. Med. (Lausanne).

[25] Nacul L, O'Boyle S, Palla L, Nacul FE, Mudie K, Kingdon CC (2020). How myalgic encephalomyelitis/chronic fatigue syndrome (ME/CFS) progresses: The natural history of ME/CFS. Front. Neurol..

[26] Augustin M, Schommers P, Stecher M, Dewald F, Gieselmann L, Gruell H (2021). Post-COVID syndrome in non-hospitalised patients with COVID-19: A longitudinal prospective cohort study. Lancet Reg. Health Eur..

[27] Townsend L, Dowds J, O'Brien K, Sheill G, Dyer AH, O'Kelly B (2021). Persistent poor health after COVID-19 is not associated with respiratory complications or initial disease severity. Ann. Am. Thorac. Soc..

[28] Knight DS, Kotecha T, Razvi Y, Chacko L, Brown JT, Jeetley PS (2020). COVID-19: Myocardial injury in survivors. Circulation.

[29] Puntmann VO, Carerj ML, Wieters I, Fahim M, Arendt C, Hoffmann J (2020). Outcomes of cardiovascular magnetic resonance imaging in patients recently recovered from coronavirus disease 2019 (COVID-19). JAMA Cardiol..

[30] Huang L, Zhao P, Tang D, Zhu T, Han R, Zhan C (2020). Cardiac involvement in patients recovered from COVID-2019 identified using magnetic resonance imaging. JACC Cardiovasc. Imaging.

[31] Cassar MP, Tunnicliffe EM, Petousi N, Lewandowski AJ, Xie C, Mahmod M (2021). Symptom persistence despite improvement in cardiopulmonary health—Insights from longitudinal CMR, CPET and lung function testing post-COVID-19. EClinicalMedicine.

[32] Tadic M, Kersten J, Nita N, Schneider L, Buckert D, Gonska B (2021). The prognostic importance of right ventricular longitudinal strain in patients with cardiomyopathies, connective tissue diseases, coronary artery disease, and congenital heart diseases. Diagnostics (Basel)..

